# Characterizing the Language Used to Discuss Death in Family Meetings for Critically Ill Infants

**DOI:** 10.1001/jamanetworkopen.2022.33722

**Published:** 2022-10-05

**Authors:** Margaret H. Barlet, Mary C. Barks, Peter A. Ubel, J. Kelly Davis, Kathryn I. Pollak, Erica C. Kaye, Kevin P. Weinfurt, Monica E. Lemmon

**Affiliations:** 1 Duke University School of Medicine, Durham, North Carolina; 2 Margolis Center for Health Policy, Duke University, Durham, North Carolina; 3 Fuqua School of Business, Duke University, Durham, North Carolina; 4 Sanford School of Public Policy, Duke University, Durham, North Carolina; 5 Department of Population Health Sciences, Duke University School of Medicine, Durham, North Carolina; 6 Department of Oncology, St Jude Children’s Research Hospital, Memphis, Tennessee; 7 Department of Pediatrics, Duke University School of Medicine, Durham, North Carolina

## Abstract

**Importance:**

Communication during conversations about death is critical; however, little is known about the language clinicians and families use to discuss death.

**Objective:**

To characterize (1) the way death is discussed in family meetings between parents of critically ill infants and the clinical team and (2) how discussion of death differs between clinicians and family members.

**Design, Setting, and Participants:**

This longitudinal qualitative study took place at a single academic hospital in the southeast US. Patients were enrolled from September 2018 to September 2020, and infants were followed up longitudinally throughout their hospitalization. Participants included families of infants with neurologic conditions who were hospitalized in the intensive care unit and had a planned family meeting to discuss neurologic prognosis or starting, not starting, or discontinuing life-sustaining treatment. Family meetings were recorded, transcribed, and deidentified before being screened for discussion of death.

**Main Outcomes and Measures:**

The main outcome was the language used to reference death during family meetings between parents and clinicians. Conventional content analysis was used to analyze data.

**Results:**

A total of 68 family meetings involving 36 parents of 24 infants were screened; 33 family meetings (49%) involving 20 parents (56%) and 13 infants (54%) included discussion of death. Most parents involved in discussion of death identified as the infant’s mother (13 [65%]) and as Black (12 [60%]). Death was referenced 406 times throughout the family meetings (275 times by clinicians and 131 times by family members); the words *die*, *death*, *dying*, or *stillborn* were used 5% of the time by clinicians (13 of 275 references) and 15% of the time by family members (19 of 131 references). Four types of euphemisms used in place of *die*, *death*, *dying*, or *stillborn* were identified: (1) survival framing (eg, *not live*), (2) colloquialisms (eg, *pass away*), (3) medical jargon, including obscure technical terms (eg, *code event*) or talking around death with physiologic terms (eg, *irrecoverable heart rate drop*), and (4) pronouns without an antecedent (eg, *it*). The most common type of euphemism used by clinicians was medical jargon (118 of 275 references [43%]). The most common type of euphemism used by family members was colloquialism (44 of 131 references [34%]).

**Conclusions and Relevance:**

In this qualitative study, the words *die*, *death*, *dying*, or *stillborn* were rarely used to refer to death in family meetings with clinicians. Families most often used colloquialisms to reference death, and clinicians most often used medical jargon. Future work should evaluate the effects of euphemisms on mutual understanding, shared decision-making, and clinician-family relationships.

## Key Points

**Question:**

What language is used to discuss death in family meetings between parents of critically ill infants and the clinical team?

**Findings:**

In this qualitative study of 33 clinician-family meetings involving 20 parents of 13 critically ill infants, the words *die*, *death*, *dying*, or *stillborn* were used in 5% of clinicians’ and 15% of family members’ references to death; 92% of references to death were euphemistic, with clinicians most often using medical jargon and family members most often using colloquialisms.

**Meaning:**

The findings suggest that use of *death* is rare in conversations about death and that clinicians often substitute *death* with medical jargon.

## Teaser

This qualitative study characterizes words used to discuss death in meetings between clinicians and parents of critically ill infants and examines differences in the way clinicians and family members refer to death.

## Introduction

When communicating with patients and families, what clinicians perceive to be small differences in language may have a profound impact. Word choice has been shown to affect patient perception of illness and decision-making.^1,2,3,4,5^ For example, when ductal carcinoma is described to women as “noninvasive cancer” or “preinvasive cancer cells,” they are more likely to prefer surgical management than when it is described as “a breast lesion” or “abnormal cells.”^6,7^ Similar results have been found for other clinical decisions.^8,9,10,11,12^

Word choice is especially important during conversations about death. Consensus guidelines emphasize the importance of clear communication, including avoiding euphemism use.^13^ Similarly, data suggest that patients and families prefer that clinicians use clear, direct language when discussing death.^14,15,16,17,18,19,20,21^ Despite these recommendations and preferences, hesitancy to reference death using the word itself is common. Studies in adult medicine have found few uses of words such as *die*, *death*, and *dying* in medical documentation,^22^ standardized patient scenarios,^23,24,25^ and recorded encounters between clinicians, patients, and families^26,27,28^ when death is discussed at all.^29^

To date, few studies have explored how discussion of death occurs in neonatal or pediatric contexts. However, clinical communication about death is common in pediatrics because most children who die each year in the US do so in a medical facility.^30^ In neonatal, pediatric, and pediatric cardiac intensive care units, children often die following a decision not to start or to discontinue life-sustaining treatment.^31,32^ Clear communication empowers decision-making in these contexts because decision-making often occurs during or shortly after family meetings.^33^ However, physician training in communication about death and dying is inadequate,^18,34,35^ and family meetings frequently lack key elements of shared decision-making.^36^

To support high-quality communication between families and clinicians of critically ill children, the ways in which death is discussed in current practice must be understood. We aimed to (1) characterize the way death is discussed during family meetings between parents of critically ill infants and the clinical team and (2) explore how discussion of death differs between clinicians and family members.

## Methods

### Data Collection

This longitudinal qualitative study was conducted at a single academic hospital in the southeast US. Patients were enrolled from September 2018 to September 2020, and infants were followed up longitudinally throughout their hospitalization. Families were approached for enrollment if their infant met the following inclusion criteria: (1) age younger than 1 year; (2) diagnosed neurologic condition; (3) hospitalization in the neonatal, pediatric, or pediatric cardiac intensive care unit at the study site; and (4) planned family meeting to discuss neurologic prognosis or starting, not starting, or discontinuing life-sustaining treatment. Parents were not enrolled if they (1) were younger than 18 years, (2) had a speech and/or hearing impairment, or (3) required translation to read or interpretation to converse in English. This study was part of a larger study^37,38,39,40^ in which parents provided written informed consent to have family meetings recorded, fill out surveys, and perform interviews; they received financial compensation for participation in the overall study at the time of interviews and survey completion. Recruitment was halted from March 16 to June 16, 2020, owing to the COVID-19 pandemic. The study protocol was approved by the Duke University Health System institutional review board. This study followed the Standards for Reporting Qualitative Research (SRQR) reporting guideline.^41^

### Identifying Discussion of Death

Family meetings occurring during enrolled infants’ hospitalizations were audio-recorded, transcribed, and deidentified before being screened for discussion of death. We defined discussion of death as discussion that included (1) mention of an infant dying owing to a current medical problem (eg, bradycardia) or diagnosis (eg, trisomy 18) or either not starting or discontinuing life-sustaining treatment, (2) mention of a prior near-death event (eg, an event requiring chest compressions), or (3) mention of a hypothetical future near-death event (eg, a conversation about the type of emergent treatment parents would or would not like their infant to receive if the infant’s heart or breathing were to stop). Family meeting transcripts that did not have any possible discussion of death (eg, a discharge planning meeting for a well infant) were screened out by 1 team member (M.H.B.). All other meetings were reviewed by 2 team members (M.H.B., M.C.B., J.K.D., or M.E.L.) to identify discussion of death in consensus.

### Codebook Development, Application, and Reconciliation

In the absence of an existing framework for discussion of death during family meetings, we developed a novel codebook using a conventional content analysis approach. The codebook was designed to encompass all discussion of death. A reference to death was defined as 1 sentence that included *die*, *death*, *dying*, or *stillborn* and/or 1 or more substitutions of such words with a euphemism. Existing linguistic literature^42^ informed the categorization of euphemisms. References using pronouns or vague nouns were only coded when the sentence including the reference and the preceding sentence did not contain any other reference to death.

Early versions of the codebook were independently pilot tested on a subset of family meetings to assess for completeness and consistency. The codebook was modified and updated through an iterative process to reach its final form (Table 1). Each transcript was coded independently by 2 members of the research team (M.H.B., M.C.B., or J.K.D). Differences in coding were resolved by third-party adjudication (M.E.L.) to achieve consensus. At times, discussions were so vague that it was challenging to be sure whether death or critical illness was being described; in these cases, we excluded ambiguous statements.

**Table 1.** Codebook for Discussions of Death Between Clinicians and Families

Reference to death	Description	Examples
*Die*, *death*, *dying*, and *stillborn*	The word itself	Clinician: “I think she would die if she got to that point.” Family member: “I don’t want to look at my child like he’s just another baby on that disease and he’s just gonna die.”
Euphemism		
Survival framing	Language about life; often a denial of the opposite (litotes)	Clinician: “We know that he’s gonna have a shorter life span.” Family member: “For us, there’s 4 outcomes with the kids, which is they don’t survive, severe disability, minor disability, no disability.”
Colloquialism	Figurative language	Clinician: “We can take her off the vent and give her to you in your arms and let her go.” Family member: “So, if you all took her off the ventilator, she would probably pass away, right?”
Medical jargon	Words or phrases with a specific meaning in medical culture that may not have the same meaning outside medicine or a concrete physiologic description without interpretation	Clinician: “If he were to code, we will not be providing chest compressions.” Clinician: “With her condition, there’s always a possibility that sometimes, she can stop breathing.”
Pronoun or vague noun	A pronoun or vague noun without its antecedent	Clinician: “Yes, it’s really hard for us to predict when it would happen.” Family member: “I know what could happen at any time, and if that does happen, I would rather it happen knowing that we got a bond.”
**Other ways death was discussed**		
Statements that imply but do not reference death		
Decision-focused language	Death is talked around or blunted by discussing medical decisions that would result in death without referencing death	Clinician: “Given what we know about her for years and years ahead, some parents have decisions, and those decisions can change about if they want the doctors to do all of the things.”
Encouraging preparation for death	Death is talked around or blunted by encouraging families to do things that only make sense if the infant is dying	Clinician: “Sometimes it’s important to do things like taking pictures with him, getting other family members to come and meet him if that’s important for you, giving blessings and christening and all that kind of stuff.”
Hoping or planning for a different outcome	Death is talked around or blunted by stating what someone wishes were true or expressing doubt about what might happen while leaving concern for death unsaid	Clinician: “I very much hope that we’re wrong and she comes rolling back in just to visit socially in 6 months to say hi and to laugh in our faces.” Family member: “If she makes it, if she pulls through and she does the time, I’m here for [baby].”
Distancing behaviors		
Generalization	Death is talked around or blunted by broadening the discussion from the infant at hand to a population of infants thought to be similar	Clinician: “Granted that she’s doing as expected for a baby with trisomy 18, there is always the possibility that these babies could have a drop in their heart rate or stop breathing.”
Burying in a monologue	Death is talked around or blunted by hiding statements about possible infant death within long technical monologues	Clinician [in the middle of an 800-word description of neuroimaging results]: “…even when babies who have this degree of injury do end up going home, they still sometimes don’t live all the way into adulthood…”

### Statistical Analysis

Parent demographic information was collected by survey at the time of enrollment. Parent race was ascertained by self-report; parents could select more than 1 race. Infant characteristics were collected by electronic medical record review after death or hospital discharge. Descriptive statistics about parents, infants, and family meetings were generated using R, version 4.0.3 (R Project for Statistical Computing). Overall code frequency and code frequency by speaker type were generated through queries in the coding software NVivo, version 12 (QSR International).

## Results

### Infant, Parent, and Meeting Characteristics

A total of 68 family meetings involving 36 parents of 24 infants were recorded. Discussion of death was present in 33 (49%) of the family meetings. The 33 family meetings that included discussion of death involved the care of 13 of the 24 total infants (54%) and 20 of the 36 total parents (56%). For all 13 infants, 1 parent identified as the infant’s mother; for 7 infants (54%), another parent was enrolled who identified as the infant’s father. Among these 13 infants, death was discussed in a median of 3 meetings (range, 1-5 meetings).

#### Infant Characteristics

In family meetings that included discussion of death, the 13 infants being discussed had a median length of hospital stay of 86 days (range, 29-217 days). The median gestational age of infants was 37 weeks (range, 23-40 weeks); 7 (54%) were female, and 6 (46%) were male (Table 2). A total of 12 infants (92%) received mechanical ventilation, and 6 (46%) required chest compressions. Five (38%) had a do-not-attempt-resuscitation order placed, and 2 (15%) died during admission.

**Table 2.** Infant and Parent Characteristics

Characteristic	Infants or parents^a^
Infants	
Sex	
Female	7/13 (54)
Male	6/13 (46)
Born prematurely	6/13 (46)
Gestational age, median (range), wk	37 (23-40)
Length of hospital stay, median (range), d	86 (29-217)
Diagnoses	
Genetic condition	8/13 (62)
Brain malformation	6/13 (46)
HIE	4/13 (31)
Seizures	7/13 (54)
Interventions	
Mechanical ventilation	12/13 (92)
Feeding tube placement	9/13 (69)
Tracheostomy	4/13 (31)
CSF diversion	2/13 (15)
Chest compressions	6/13 (46)
DNAR order	5/13 (38)
Death during admission	2/13 (15)
**Parents**	
Mother	13/20 (65)
Father	7/20 (35)
Age, median (range), y	28.5 (19-43)
Race^b^	
Asian	2/20 (10)
Black	12/20 (60)
Multiracial	0/20 (0)
White	8/20 (40)
Hispanic ethnicity	0/20 (0)
Level of education	
Less than high school	2/20 (10)
High school or GED	6/20 (30)
Some college	7/20 (35)
Associate’s or technical degree	0/20 (0)
Bachelor’s degree	1/20 (5)
Graduate or professional degree	4/20 (20)
Annual family income, $^c^	
<25 000	7/12 (58)
25 000-34 999	2/12 (17)
35 000-49 999	0/12 (0)
50 000-74 999	1/12 (8)
75 000-99 999	0/12 (0)
100 000-149 999	1/12 (8)
>150 000	1/12 (8)
Self-reported spirituality	
Strongly	10/20 (50)
Somewhat	9/20 (45)
Slightly	0/20 (0)
Not at all	1/20 (5)

Abbreviations: CSF, cerebrospinal fluid; DNAR, do not attempt resuscitation; GED, General Educational Development; HIE, hypoxic ischemic encephalopathy.

^a^ Data are presented as number/total number (percentage) of infants or parents unless otherwise indicated.

^b^ Numbers sum to more than 20 because some parents self-identified as more than 1 race.

^c^ As reported by mothers to avoid overrepresentation of families with 2 parents enrolled; 1 mother declined to provide this information.

#### Parent Characteristics

Of the 20 parents (median age, 28.5 years [range, 19-43 years]) involved in family meetings that included discussion of death, 2 (10%) identified as Asian, 12 (60%) as Black, and 8 (40%) as White (some selected more than 1 race) (Table 2). Most parents (13 [65%]) involved in discussion of death identified as the infant’s mother. Fifteen (75%) reported that their highest level of education was some college, high school or General Educational Development, or less. To avoid overrepresentation among families with 2 parents enrolled, we only included family income data reported by mothers; 7 of 12 mothers (58%) reported that the annual family income was less than $25 000 (1 mother [8%] declined to provide this information).

#### Meeting Characteristics

Family meetings that included discussion of death had a median length of 44 minutes (range, 21-85 minutes) (Table 3). A median of 8 people (range, 4-19 people) participated in these meetings, most of whom were clinicians (median, 6 per meeting; range, 3-12 per meeting).

**Table 3.** Meeting Characteristics

Characteristic	Meetings (N = 33)^a^
Length of meeting, median (range), min	44 (21-85)
Participants, median (range), No.	
Family members	2 (1-7)
Clinicians	6 (3-12)
Total	8 (4-19)
Family members present	
Mother	33 (100)
Father	20 (61)
Grandparent	12 (36)
Clinicians present	
Attending physician	32 (97)
Fellow	19 (58)
Resident	8 (24)
Nurse practitioner	29 (88)
Social worker	27 (82)
Case manager	4 (12)
Bedside nurse	9 (27)
Services present	
Neonatology	31 (94)
Palliative care	24 (73)
Neurology	22 (67)
Proportion of meeting spent discussing death,^b^ median (range), %	11 (1-61)

^a^ Data are presented as the number (percentage) of meetings unless otherwise indicated.

^b^ Defined as the number of words coded as discussion of death divided by the total words in the meeting transcript.

### Use of *Die*, *Death*, *Dying*, and *Stillborn*

The words *die*, *death*, *dying*, and *stillborn* were used a total of 32 times in 406 references to death (8%) and were present in 15 of the 33 family meetings that included discussion of death (45%). Whereas most words during discussions of death were spoken by clinicians (26 308 of 36 130 words [73%]), family members were responsible for most uses of *die*, *death*, *dying*, or *stillborn* (19 of 32 uses [59%]). When referencing death, family members were more likely to use *die*, *death*, *dying*, or *stillborn* than were clinicians (19 of 131 total references by families [15%] vs 13 of 275 total references by clinicians [5%]). Among family members, mothers were most likely to use *die*, *death*, *dying*, or *stillborn* (17 of 19 total references by families [89%]). Among clinicians, palliative care clinicians were most likely to use these terms (7 of 13 total references by clinicians [54%]).

Many uses of *die*, *death*, *dying*, or *stillborn* by clinicians followed a parent’s request for clarity. For example, the following exchange took place after a clinician’s 350-word description of bradycardic episodes in an infant with a congenital brain anomaly:

Mom: What you’re saying, I mean, I want you to say it.

Intensive care unit fellow: So, I’m going to say it. I will say it. I’ll say it. I’m concerned that at some point her heart rate may go all the way down and not come back up and she’ll die.

Many uses of *die*, *death*, *dying*, or *stillborn* by family members involved recounting something that they had previously been told:

Dad: When [mom] first gave birth to [baby], there was a pretty rude lady that walked in the room and said that she’s probably gonna die.

### Substitution of *Die*, *Death*, *Dying*, or *Stillborn* With a Euphemism

**Figure 1.**
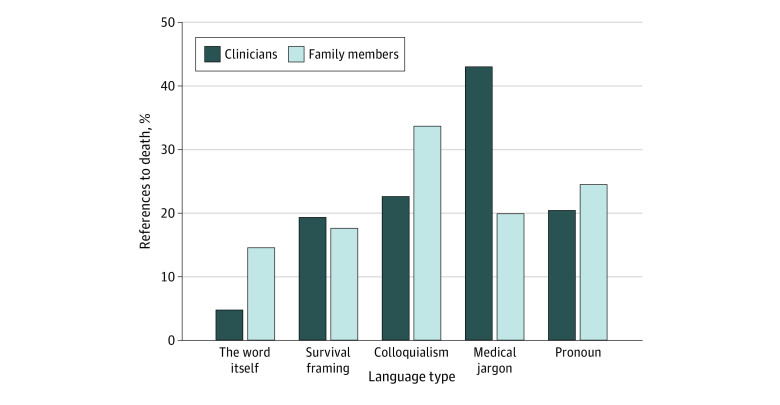
Prevalence of Types of Language Used by Clinicians and Family Members to Refer to Death Proportions represent relative frequencies; for each language type, the number of uses was scaled by the total number of references to death made by each group (275 for clinicians, 131 for family members). “The word itself” refers to *die*, *death*, *dying*, or *stillborn.*

Clinicians and family members replaced *die*, *death*, *dying*, and *stillborn* with a euphemism most of the time (374 of 406 total references [92%]) but differed in the types of euphemisms they used (Figure 1). We identified 4 types of euphemisms used to reference death: medical jargon, colloquialisms, pronouns and vague nouns, and survival framing.

#### Medical Jargon

The most common type of euphemism was medical jargon, in which death was obscured by technical terms or concrete physiologic descriptions. Medical jargon accounted for 144 of all 406 references to death (36%), including 118 of the 275 references made by clinicians (43%).

Medical jargon took 2 major forms. In the first, death was described using technical words or phrases with connotations that may not be clear to nonclinicians. This included shorthand for past or future near-death events, such as *event*, *episode*, *code*, or *arrest*, as well as phrases such as *ultimately fatal*, *guarded prognosis*, or *redirect care to comfort measures*.

The second form of medical jargon was physiologic circumlocution, in which a concrete description of death was given without interpretation. This sometimes overlapped with use of technical words or phrases but often involved plain language:

Clinician: It’s possible that one time, when this heart rate drop does happen, it could be an irrecoverable drop. If that makes sense.

#### Colloquialisms

The next most common type of euphemism was colloquialisms, in which *death* was replaced with figurative language. Colloquialisms accounted for 106 of all 406 references to death (26%) and were the most common type of euphemism used by family members (44 of 131 references [34%]).

There were multiple distinct types of colloquialisms; death was abstracted using a variety of metaphors, including a journey to the afterlife, losing a fight or battle, and running out of time. The most common colloquialisms were *pass away* and *not make it*.

#### Pronouns and Vague Nouns

The next most common type of euphemism was pronouns and vague nouns, which accounted for 88 of all 406 references to death (22%). Clinicians and family members referenced death using pronouns and vague nouns at similar rates.

Ten different pronouns and vague nouns were used without antecedents; the most common were *it*, *that*, *what*, *this*, and *something*. For example, the following statement occurred during a discussion about discontinuing life-sustaining treatment for an infant with a congenital brain malformation:

Clinician: Would you rather be here, holding him, with your family, surrounded by all your loved ones, when that happens?

#### Survival Framing

The final type of euphemism that we identified was survival framing, which accounted for 76 of all 406 references to death (19%). Clinicians and family members referenced death using survival framing at similar rates.

In survival framing, language about life was used to reference death. These statements were commonly what linguists call a litotes, or denial of the opposite, such as *don’t live* or *not survive*. Other times, this language was more centered on survival rates or length of life, as in “we don’t know how long she may live” or “his life is gonna be short.”

### Other Ways in Which Death Was Discussed

#### Statements That Left the Potential for Death Implied

In decision-focused language, decisions that might result in death, such as discontinuing life-sustaining treatment or placing a do-not-attempt-resuscitation order, were discussed without any reference to death. This type of language was used in 21 of the 33 family meetings that included discussion of death (64%) and was often initiated by clinicians. In 11 of these meetings (52%), there were no uses of *die*, *death*, *dying*, or *stillborn*.

In encouraging preparation for death, statements that encouraged families to prepare for death were made without referencing death. These statements often took the form of logistical arrangements for legacy-building services or suggestions to parents to enjoy the remaining time they had with their infant. This type of language was used in 16 of the 33 family meetings in which death was discussed (48%) and was typically initiated by clinicians. In 6 of these meetings (38%), there were no uses of *die*, *death*, *dying*, or *stillborn*.

In hoping or planning for a different outcome, speakers made statements about what they wished was true, leaving concern for the possibility of infant death unsaid. For example, instead of directly disagreeing with a family member’s optimistic statement, a clinician might say, “We always join you in hoping for a miracle.” This type of language occurred in 10 of the 33 family meetings that included discussion of death (30%). In 6 of these meetings (60%), there were no uses of *die*, *death*, *dying*, or *stillborn*.

#### Behaviors That Distanced Discussion of Death From the Infant

Some statements about death were generalized by using an indefinite pronoun (most commonly the generic *you*) or by discussing all infants with the same diagnosis. For example, instead of directly voicing concern about the infant being discussed, a clinician might say, “Even when babies who have this degree of injury do end up going home, they still sometimes don’t live all the way into adulthood.” This behavior occurred in 43 of all 406 references to death (11%) and was almost exclusively done by clinicians (41 of 43 [95%]).

In addition, the possibility of death was sometimes blunted by being disclosed in the middle of a monologue or by being immediately followed by an unrelated question. Thirty-six of all 406 references to death (9%) occurred in a monologue. This behavior was unique to clinicians.

## Discussion

In this study, we developed and applied a novel analytic framework to characterize the language used to discuss death in meetings between families of critically ill infants with neurologic conditions and the clinical team. The words *die*, *death*, *dying*, or *stillborn* were used in 45% of meetings in which death was discussed and accounted for 8% of references to death. Clinicians referenced death more than twice as often as family members, and clinician statements accounted for 73% of words spoken during discussion of death. Despite these findings, clinicians used *die*, *death*, *dying*, or *stillborn* less often than did family members. Whereas family members often used mutually understood colloquialisms to reference death, clinicians most often used medical jargon. Although research on this subject is limited, our results are consistent with studies in adult settings that showed high rates of vague, euphemistic language when discussing death.^26,27,28^ We also identified several ways in which death was talked around or blunted, some of which have been partially described in previous work.^28,29^ We highlight 3 important implications of word choice in this context.

**Figure 2.**
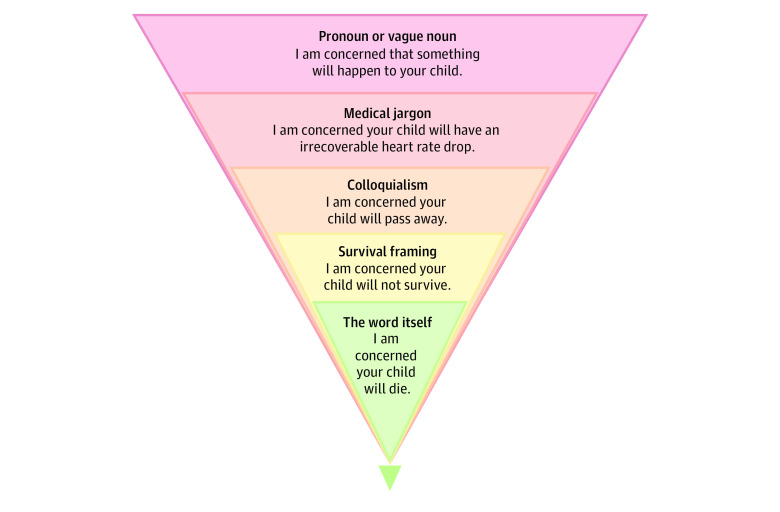
Proposed Framework for Conceptualizing the Clarity of 5 Common Ways to Reference Death The narrowest and most precise type of reference, use of *die*, *death*, *dying*, or *stillborn* (“the word itself”), is most clear, whereas euphemisms leave more room for misunderstanding.

Some words may better facilitate patient and family understanding of clinical information than others, empowering informed decision-making. During and after family meetings, family members often make high-stakes decisions on behalf of their loved ones.^33^ These decisions may be about life and death,^43,44,45^ or they may be more logistical, such as arranging spiritual rituals, calling other family members to the bedside, and taking time off work. Withholding clear communication about a patient’s status may increase the risk that stakeholders misinterpret information that is central to decision-making.^46^ Although our work did not directly evaluate the comparative clarity of different ways to reference death, our results raise questions about what language is most clear (Figure 2). Some euphemism types, such as survival framing or commonly understood colloquialisms, may be more likely to be universally understood than others. In particular, parents and families cite jargon as a barrier to clear communication^18,19,20,47^ and overestimate their ability to understand such terms.^48^ Classification systems such as the one in Figure 2 can establish a framework for understanding and discussing word choice, priming clinicians to notice and reflect on the language they hear and use. Empirically evaluating the perceived clarity of euphemism types and their effects on shared decision-making should be a priority for future study and should be used to inform interventions for improving communication in this context.

Word choice represents an opportunity for clinicians to honor patient and family preferences for communication. Interviews with patients and family members suggest that they want clinicians to use clear language^14,15,16,17,18,19,20^ even when it is hard to hear in the moment.^21^ However, many clinicians worry that words like *die*, *death*, or *dying* may be perceived as harsh^16^ and prefer to match the language used by patients and families.^17,49^ Our design did not allow for direct assessment of family preferences, including whether acknowledging death using the word itself a single time or mirroring the language used by family members is sufficient. Given that we found that most references to death made by family members were euphemistic, future work should prioritize assessing family preferences for language use.

In addition, although clinicians may avoid using words like *die*, *death*, or *dying* in an attempt to maintain rapport, previous work suggests that ambiguous or euphemistic language may erode trust in the clinical team.^18,21,47^ Future work should investigate how word choice during discussions of death impacts perceived transparency and therapeutic alliance. Although this study focused on language about death, other topics such as potential for future disability are often discussed during family meetings in this population. Our analytic approach offers a strategy to analyze other high-stakes language use in future studies.

### Limitations

This study has limitations. The results represent data from a single institution. Although enrolled families were racially diverse, the exclusion of families who did not speak English represents an important gap in the study population. Because we were able to study only what was said, questions about speaker motivation, listener understanding, and the effects of language choice on decision-making remain unanswered. Likewise, we did not code statements that were so vague they could not be definitively identified as relating to death. In addition, family meetings represent an isolated communication venue; future work should characterize how clinicians discuss death in other clinical settings and with other patient populations.

## Conclusions

In this study, discussion of death during meetings between clinicians and parents of critically ill infants rarely involved use of *die*, *death*, *dying*, or *stillborn*. Whereas family members most often referred to death using colloquial euphemisms, clinicians most often referred to death using medical jargon, statements that left death implied, and communication behaviors that distanced references to death from the patient being discussed. Future work is needed to explore how word choice affects shared decision-making and therapeutic alliance.
